# Supplementary material for: Lilac (Syringa oblata) genome provides insights into its evolution and molecular mechanism of petal color change
Source: Commun Biol. 2022 Jul 9;5:686. doi: 10.1038/s42003-022-03646-9 (PMC9271065; doi:10.1038/s42003-022-03646-9)
Supplement: Supplementary file 2 — Supplementary Information [file 42003_2022_3646_MOESM2_ESM.pdf]

Supplementary Figures

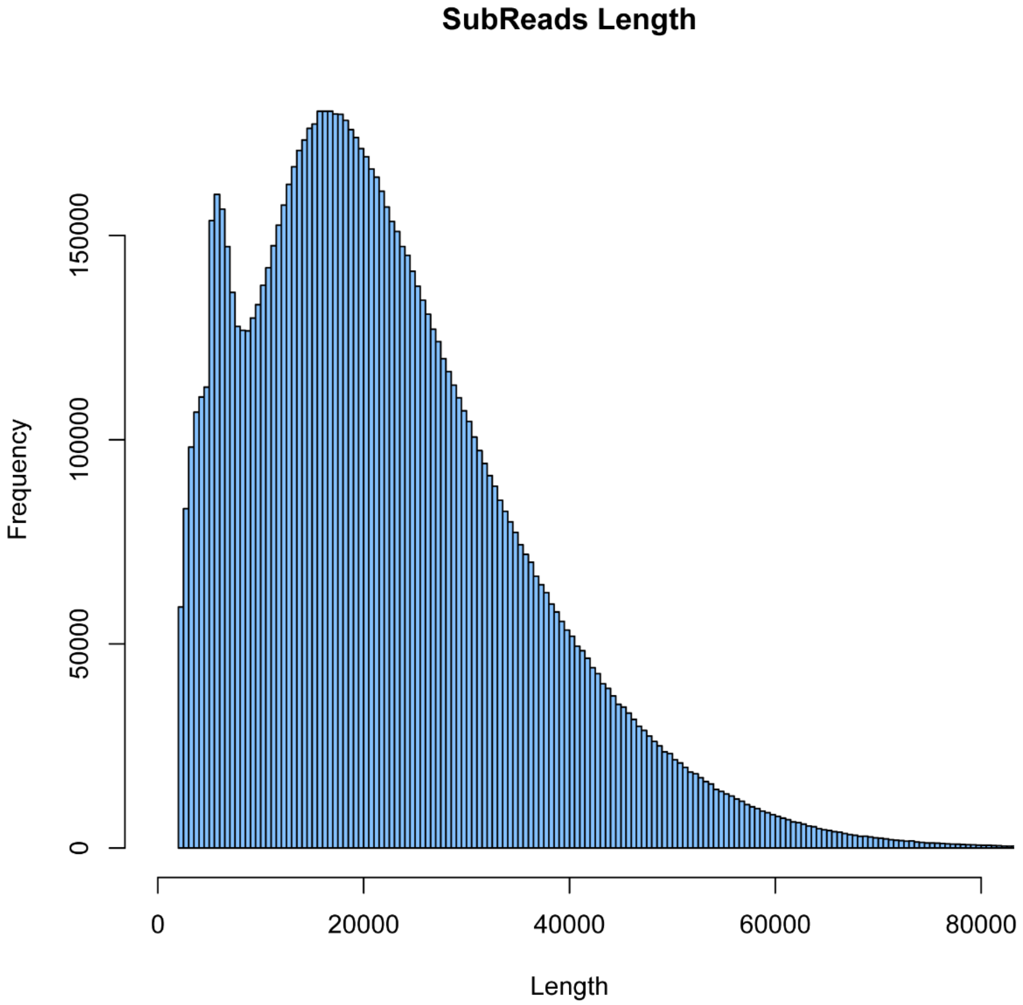

Supplementary Figure 1. Subreads length distribution of ONT sequencing data.

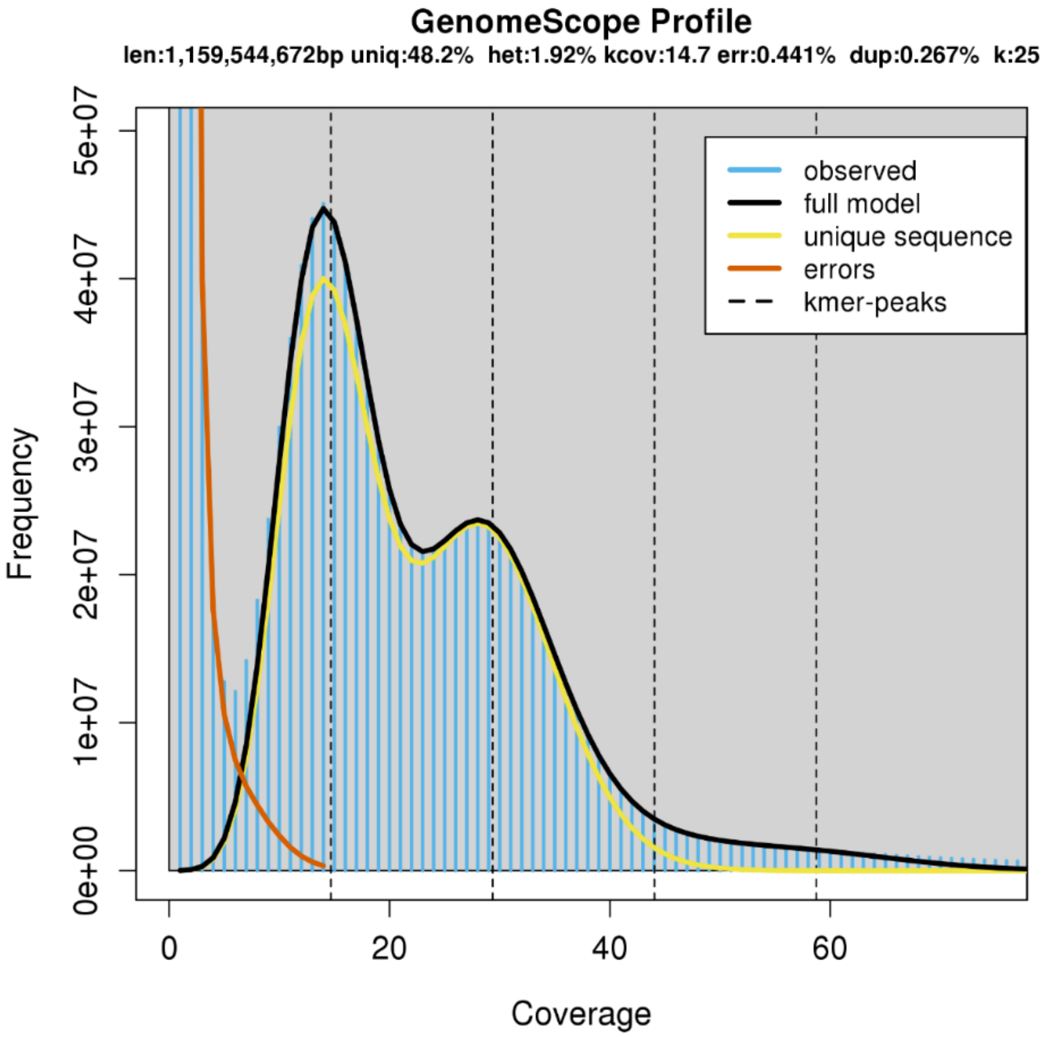

Supplementary Figure 2. The 25-mer analysis to estimate the lilac genome size.

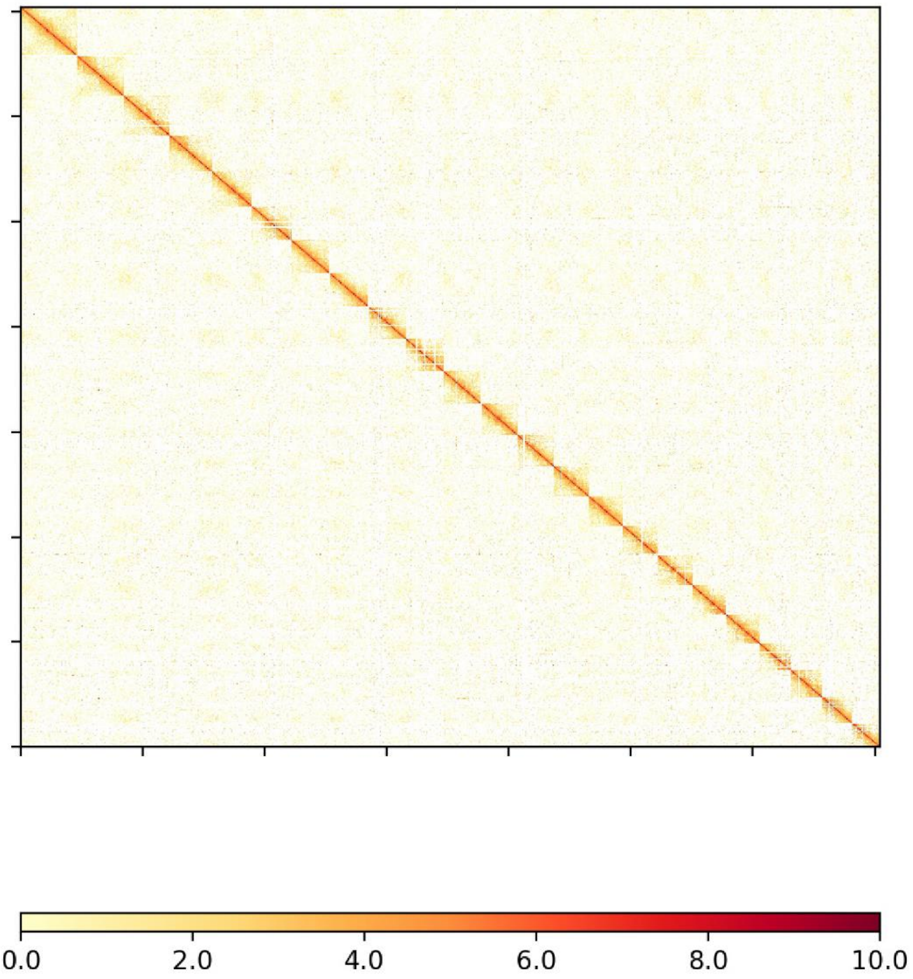

Supplementary Figure 3. Genome-wide analysis of chromatin interactions in the genome based on Hi-C data.

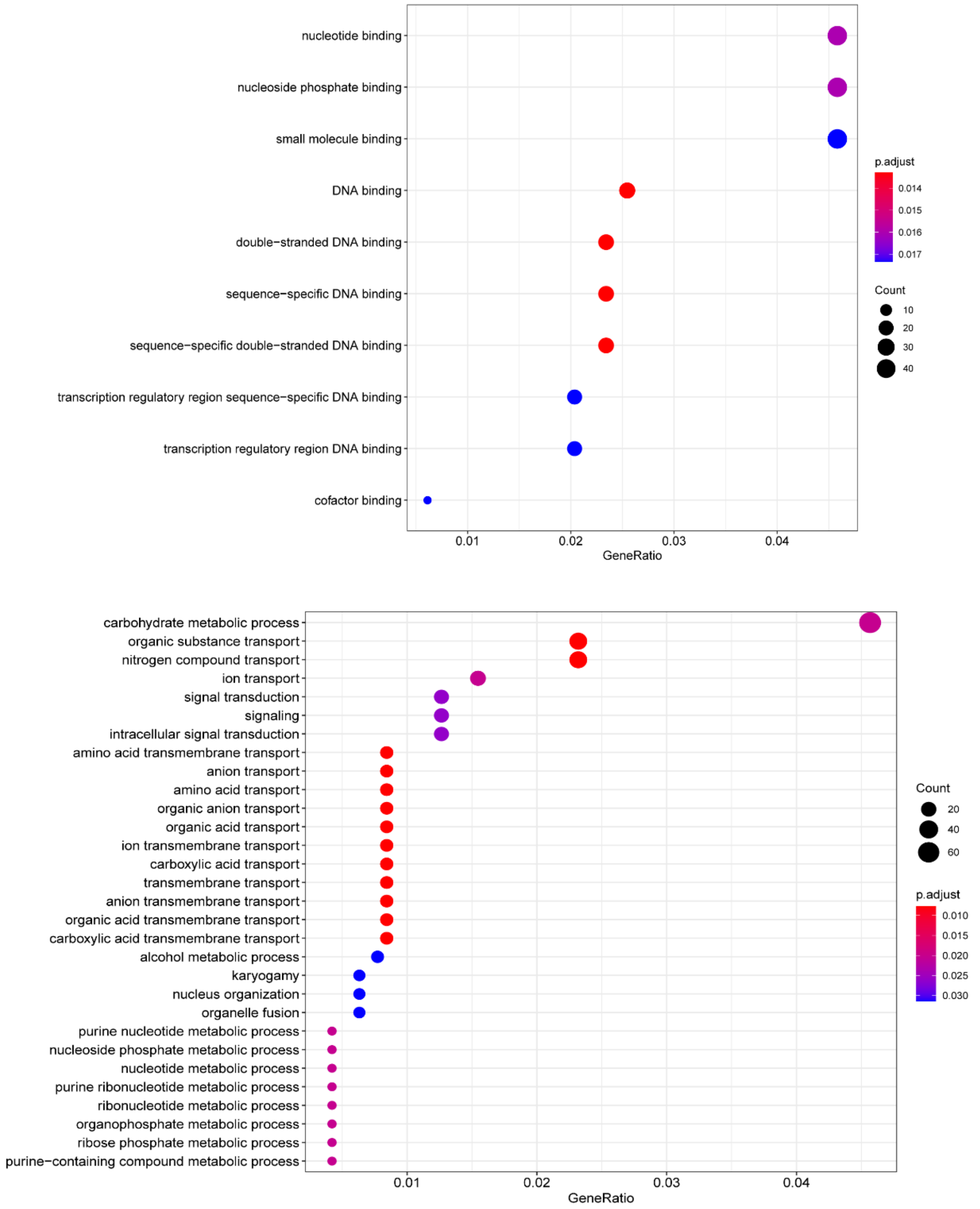

Supplementary Figure 4. GO functions (molecular function and biological process) enrichment of expanded genes in lilac.

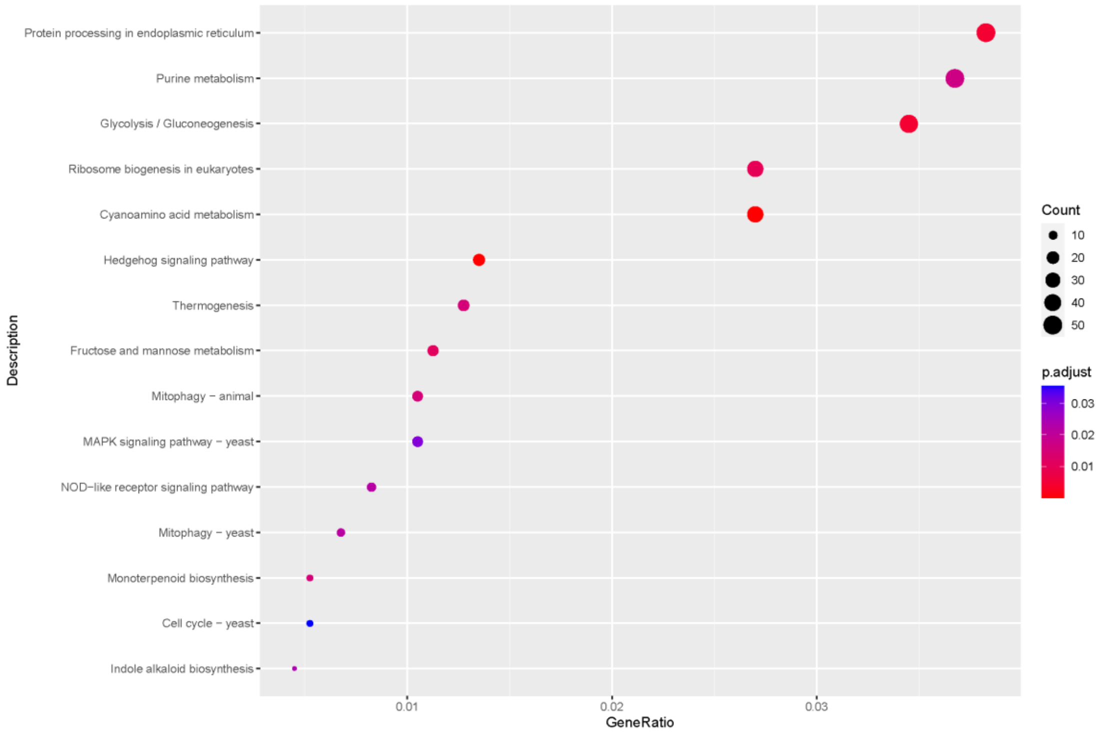

Supplementary Figure 5. KEGG pathway enrichment of expanded genes in lilac.

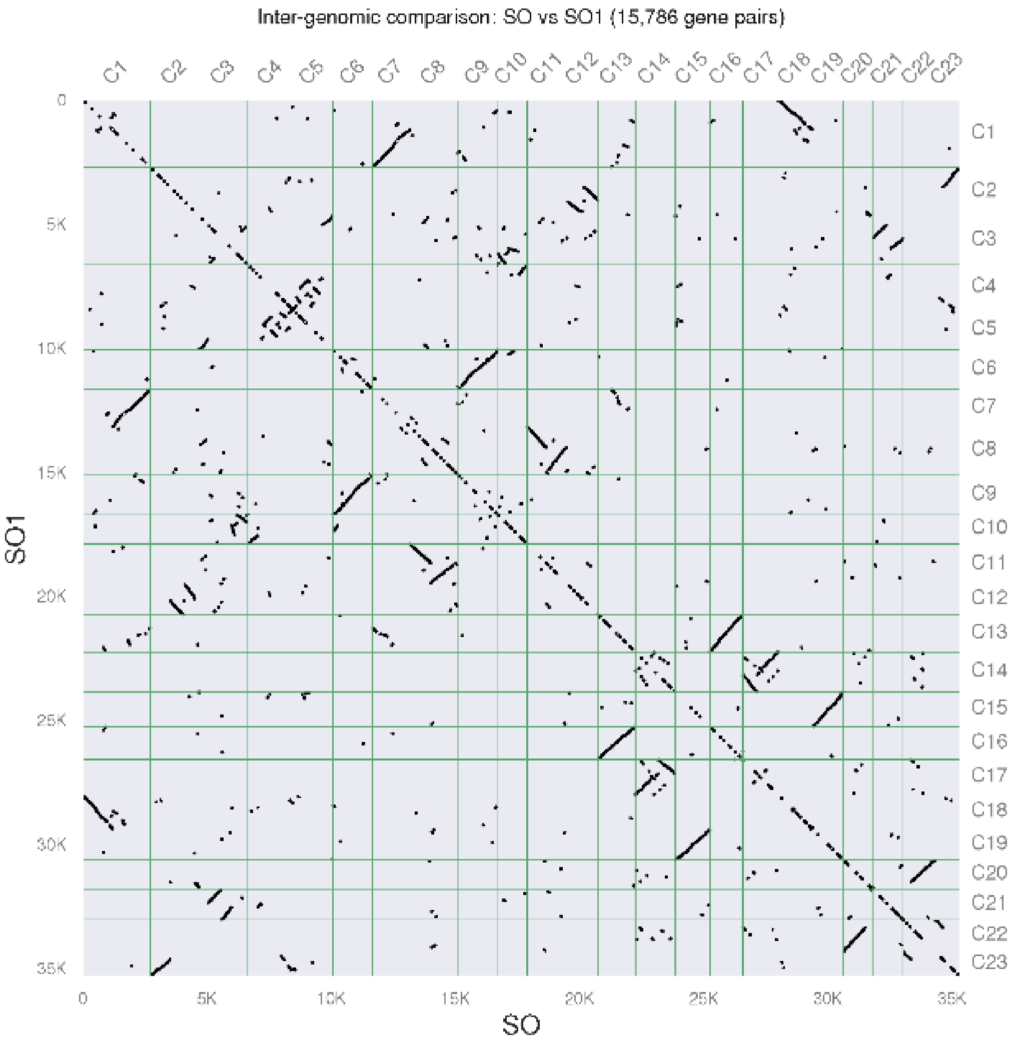

Supplementary Figure 6. The dot plot of paralogs in the *S. oblata* (SO) genome.

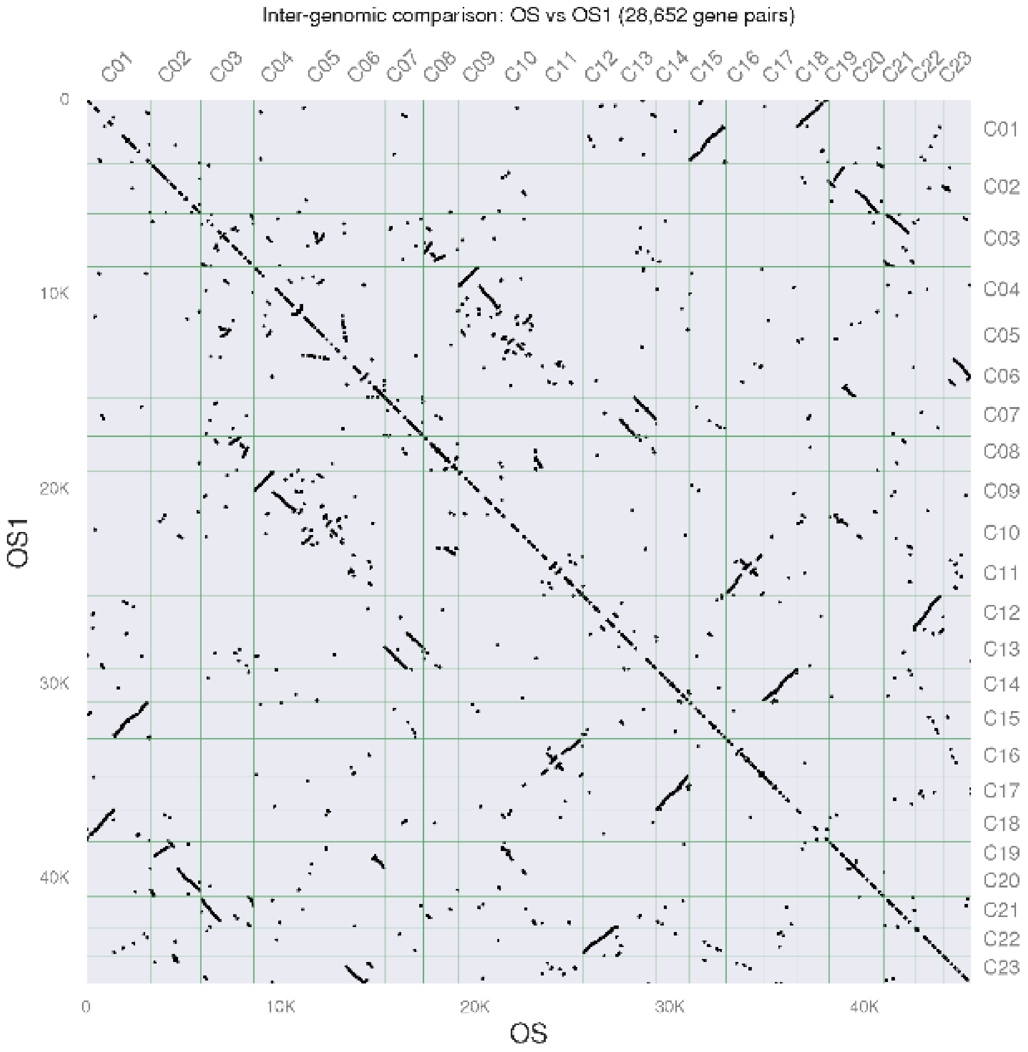

Supplementary Figure 7. Dot plot of paralogs in the *O. fragrans* (OS) genome.

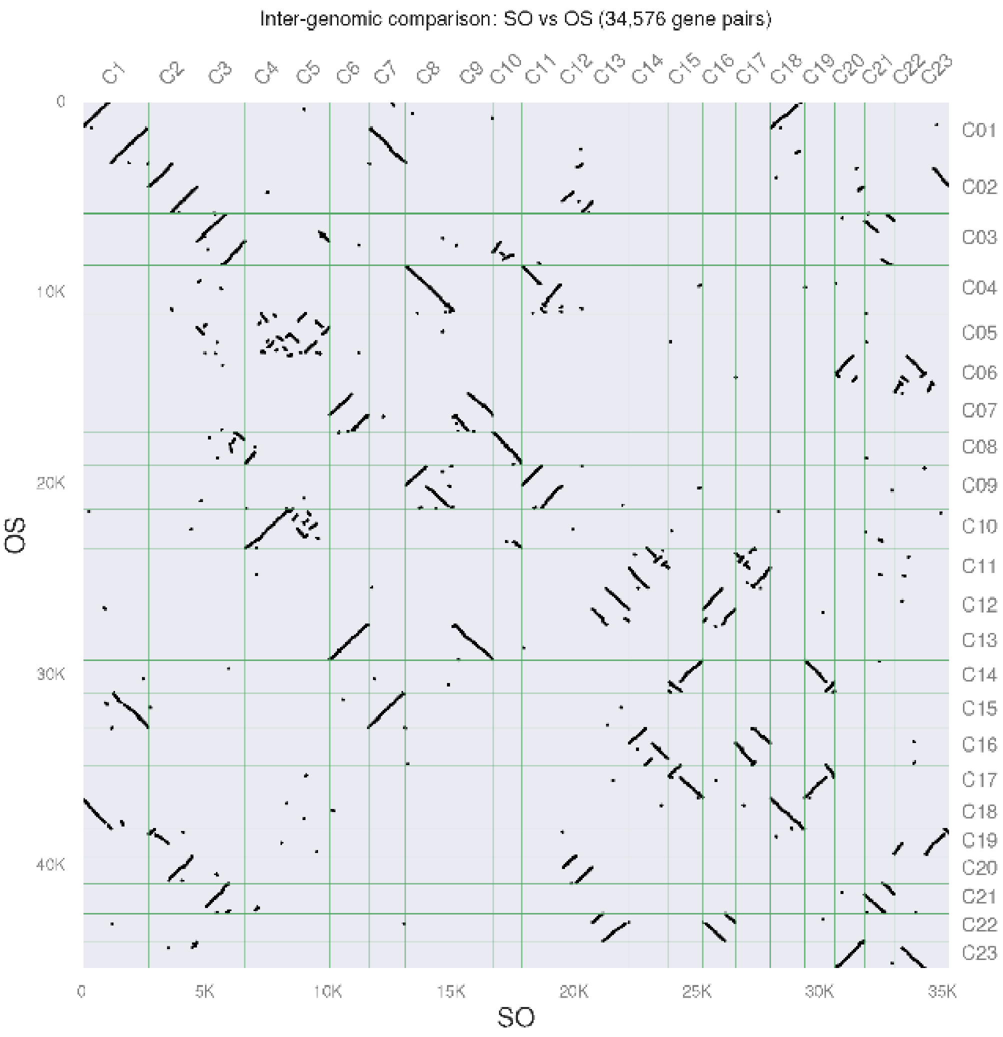

Supplementary Figure 8. The dot plot of paralogous blocks between *S. oblata* and *O. fragrans* genome.

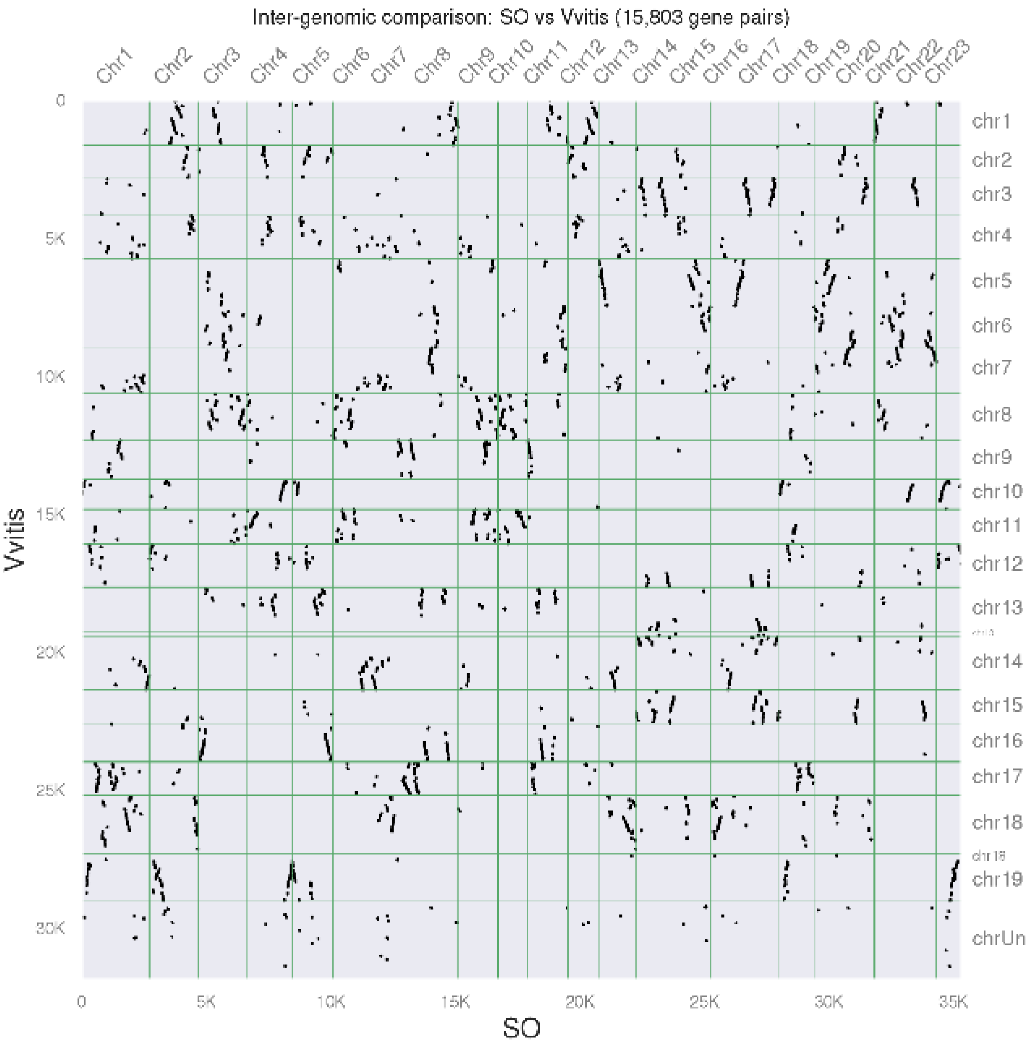

Supplementary Figure 9. The dot plot of paralogous blocks between *S. oblata* and *V. vinifera* genome.

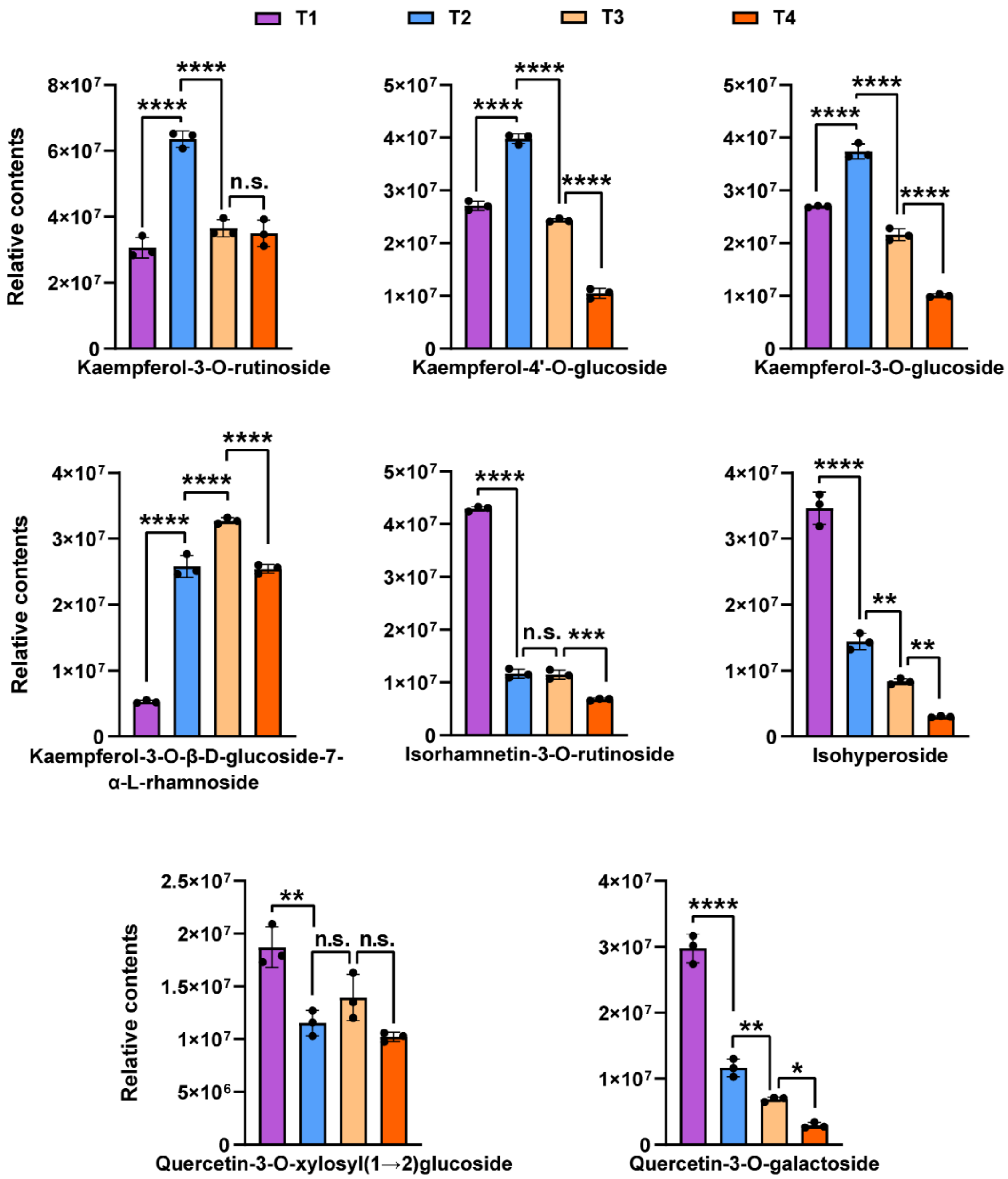

Supplementary Figure 10. Relative contents of top10 metabolites (except Dp3Ru and Cy3Ru in Fig.4b) of lilac petals at four flower opening stages (T1-T4). Data are presented as mean ±SE, n=3 biologically independent samples; statistical significance was calculated by one-way ANOVA with multiple comparisons between adjacent stages, and was indicated by asterisks, in which n.s. (no significance), $p < 0.05$ (*), $p < 0.01$ (**), $p < 0.001$ (***), and $p < 0.0001$ (****).

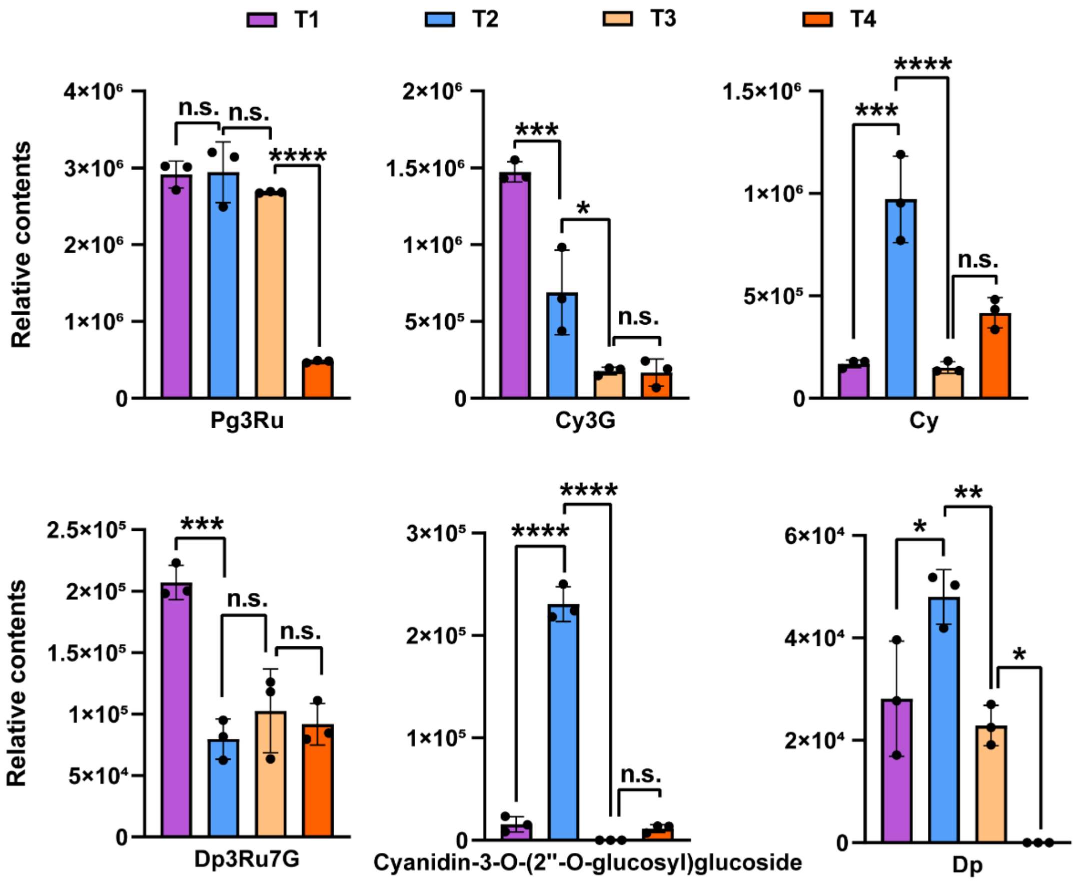

Supplementary Figure 11. Relative contents of all 9 anthocyanidins detected of lilac petals (except Dp3Ru, Cy3Ru and Pt3Ru in Fig.4b) at four flower opening stages (T1-T4). Data are presented as mean ±SE, n=3 biologically independent samples; statistical significance was calculated by one-way ANOVA with multiple comparisons between adjacent stages, and was indicated by asterisks, in which n.s. (no significance), $p < 0.05$ (*), $p < 0.01$ (**), $p < 0.001$ (***), and $p < 0.0001$ (****).

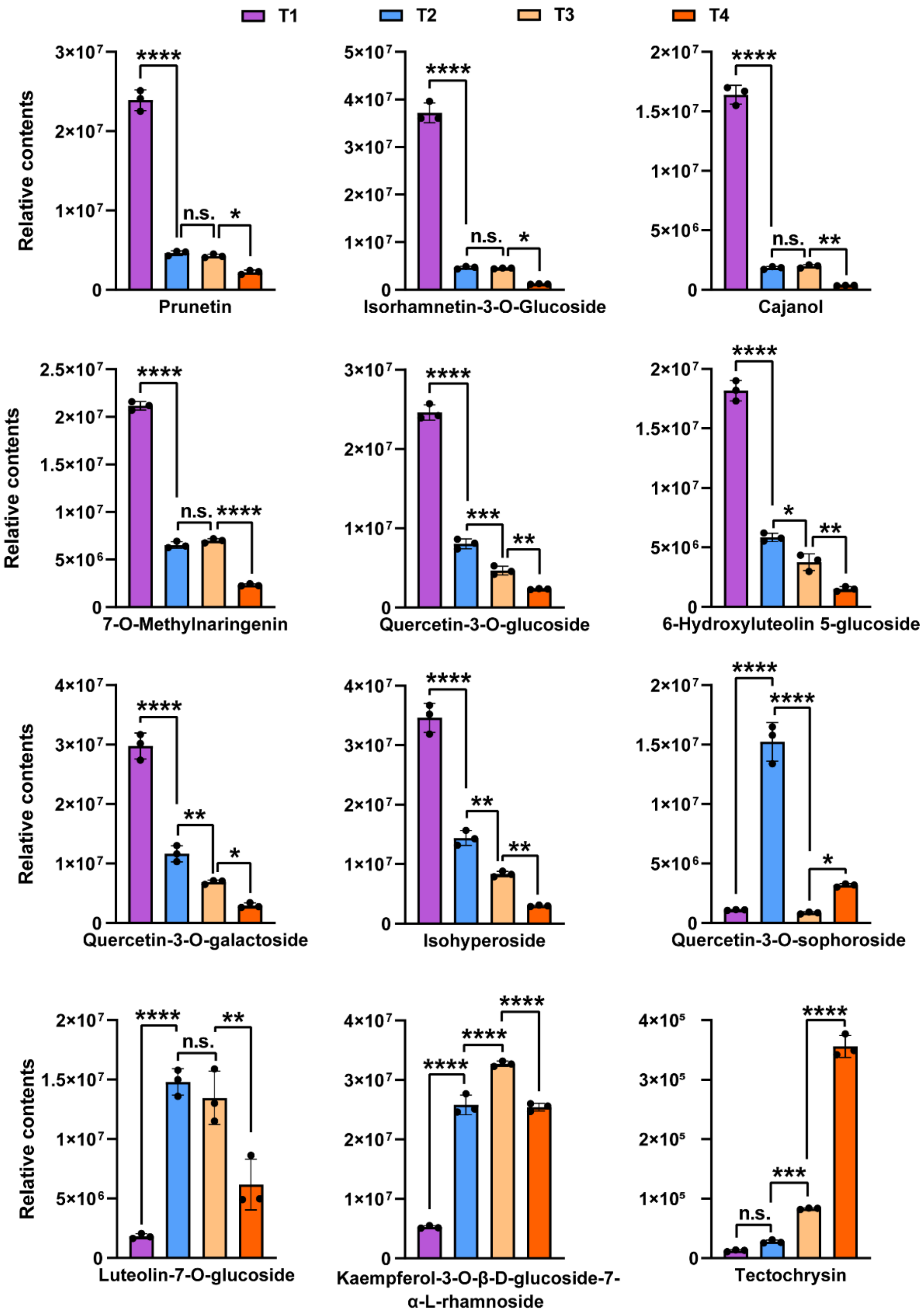

Supplementary Figure 12. Relative contents of 12 differential metabolites of lilac petals used in PCA biplot (Fig. 4c) at four flower opening stages (T1-T4). Data are presented as mean ±SE, n=3 biologically independent samples; statistical significance was calculated by one-way ANOVA with multiple comparisons between adjacent stages, and was indicated by asterisks, in which n.s. (no significance), $p < 0.05$ (*), $p < 0.01$ (**), $p < 0.001$ (***), and $p < 0.0001$ (****).

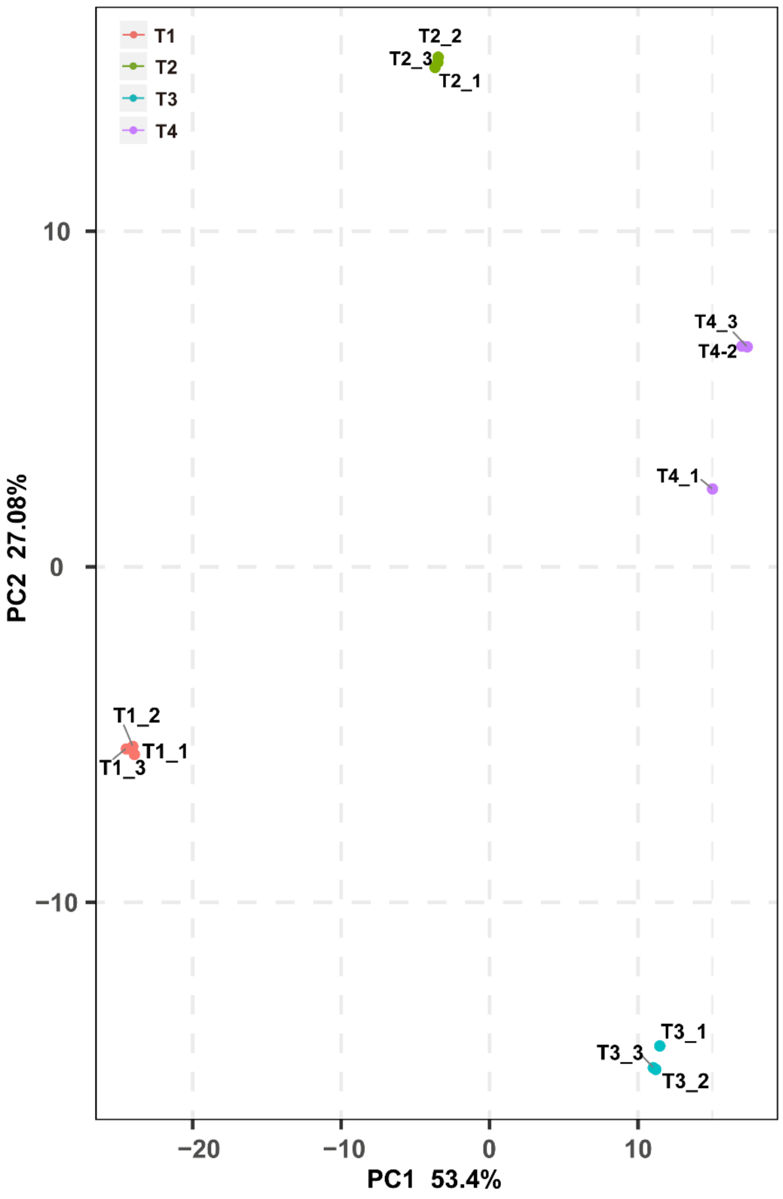

Supplementary Figure 13. PCA shows clustering of samples based on the similarity of gene expression and metabolite content at four flower opening stages (T1-T4).

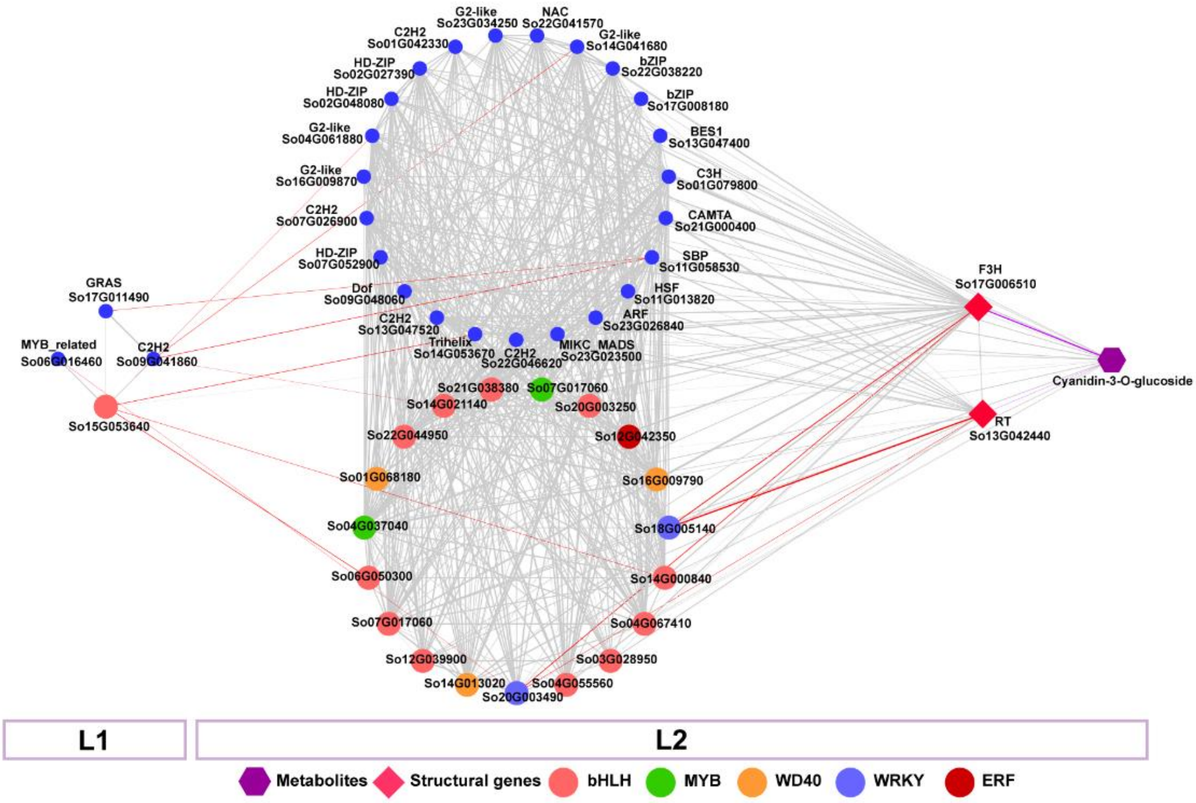

Supplementary Figure 14. Sub-network for flavonoids biosynthesis pathways at the early stage of flowering. Resolved one of the biosynthesis of Cy3G hierarchical regulation for *F3H* and *RT* gene involved. From right to left, the network consists of metabolite (Cy3G in L2), structural gene (*F3H* and *RT* in L2), TFs that play a direct regulatory role (L2), and upstream regulatory TFs (L1).

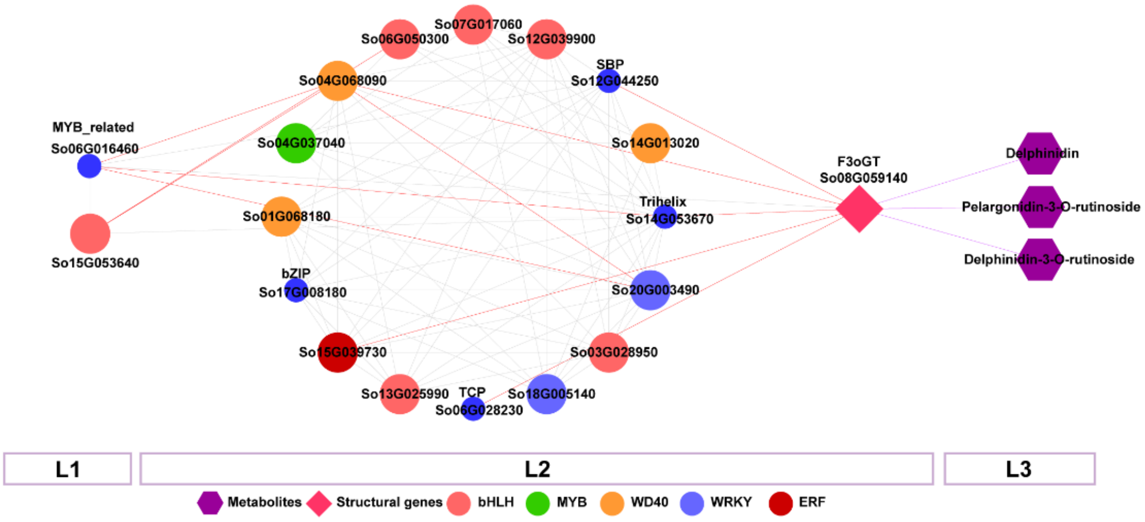

Supplementary Figure 15. Sub-network for flavonoids biosynthesis pathways at the early stage of flowering. Resolved one of the biosynthesis of Dp, Dp3Ru, Pg3Ru and hierarchical regulation for *F3oGT* gene involved. From right to left, the network consists of metabolite (L3), structural gene (*F3oGT* in L2), TFs that play a direct regulatory role (L2), and upstream regulatory TFs (L1).

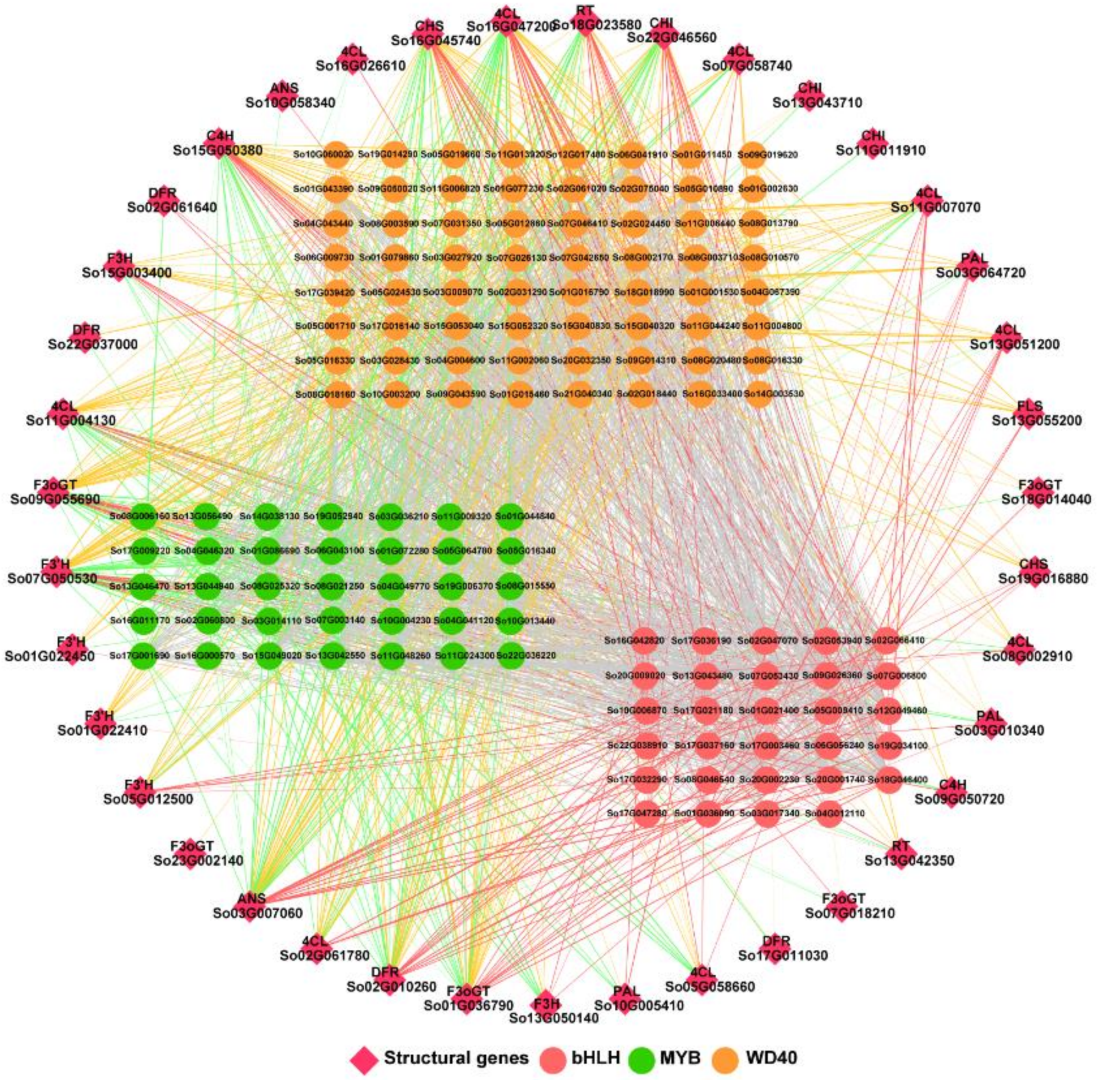

Supplementary Figure 16. Sub-network for flavonoids biosynthesis containing only the three families of TFs *MYB* (green), *bHLH* (pink), and *WD40* (orange) at the transition stage of flowering. The structural genes were linked to *bHLH*, *MYB* and *WD40* in red, green, and orange lines, respectively.

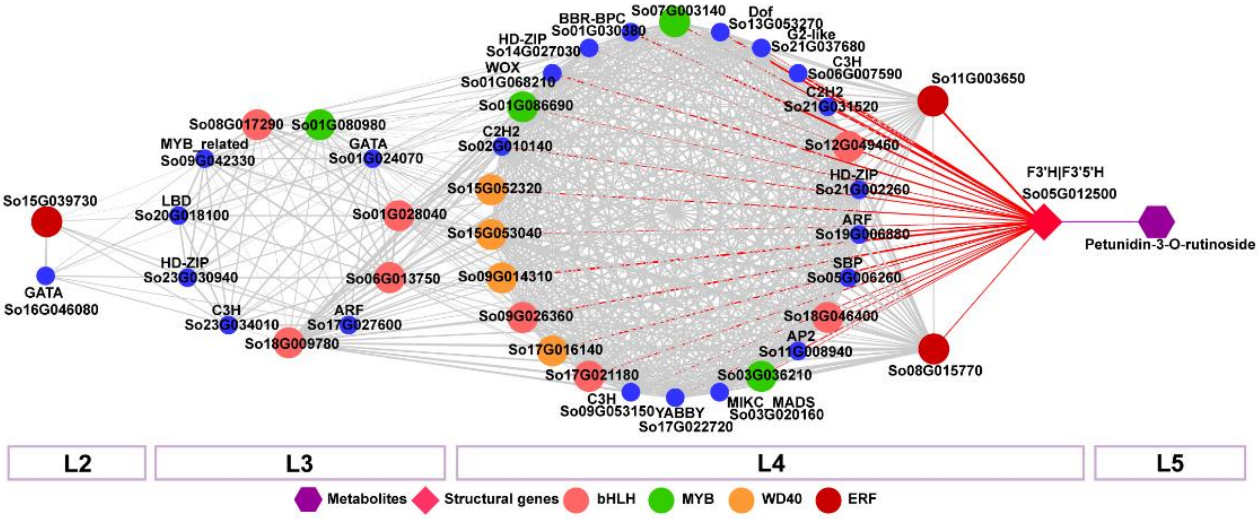

Supplementary Figure 17. Sub-network for flavonoids biosynthesis pathways at the transition stage of flowering. Resolved one of the biosynthesis of Pt3Ru and hierarchical regulation for *F3'H*|*F3'5'H* gene involved. From right to left, the network consists of metabolite (Pt3Ru in L5), structural gene (*F3'H*|*F3'5'H* in L4), TFs that play a direct regulatory role (L4), and upstream regulatory TFs (L3 and L2).

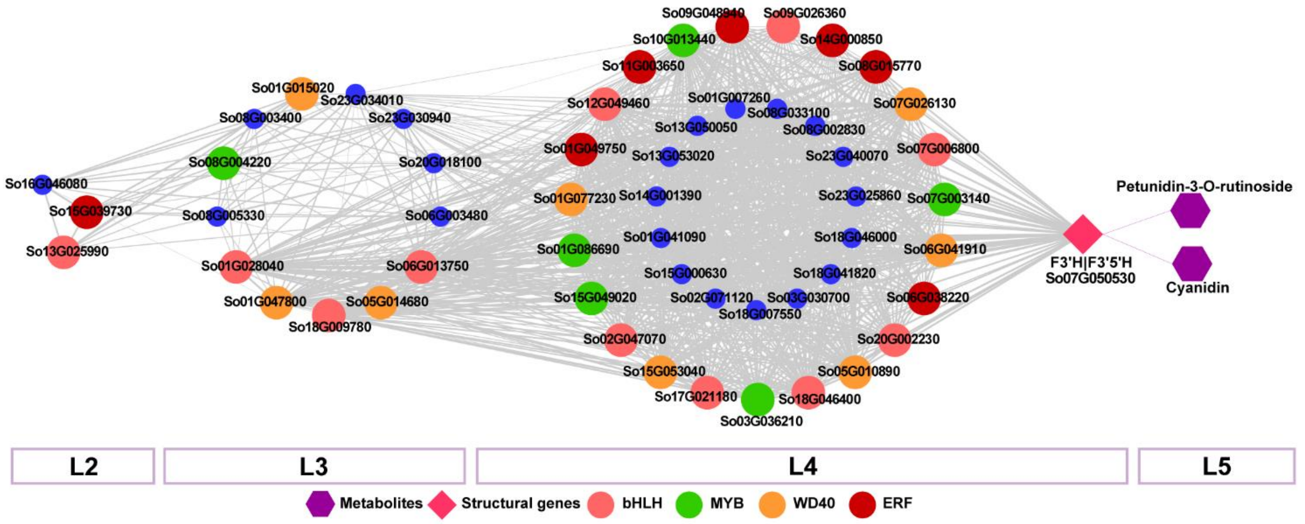

Supplementary Figure 18. Sub-network for flavonoids biosynthesis pathways at the transition stage of flowering. Resolved one of the biosynthesis of Pt3Ru and Cy and hierarchical regulation for *F3'H*|*F3'5'H* gene involved. From right to left, the network consists of metabolite (Pt3Ru and Cy in L5), structural gene (*F3'H*|*F3'5'H* in L4), TFs that play a direct regulatory role (L4), and upstream regulatory TFs (L3 and L2).

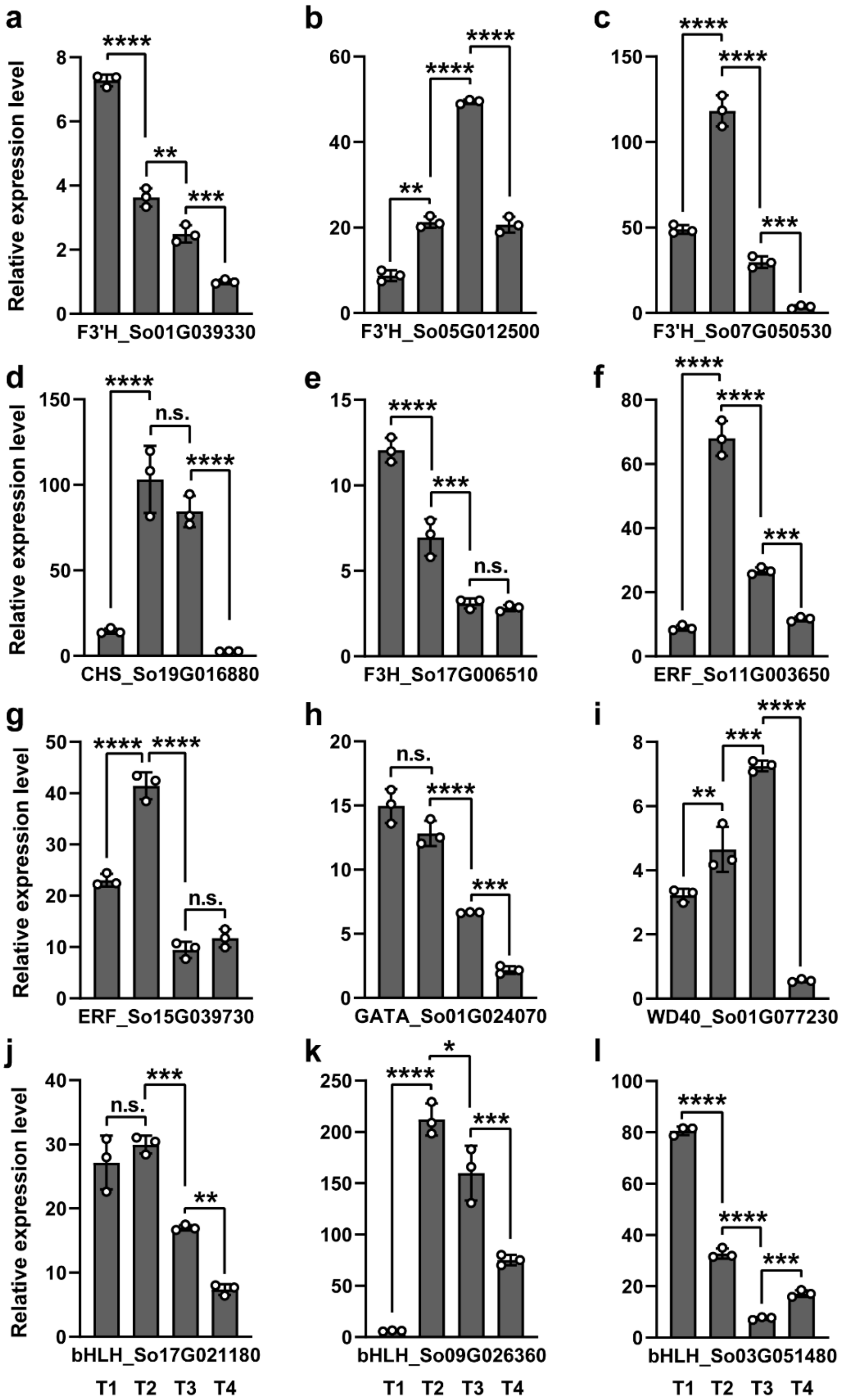

Supplementary Figure 19. QRT-PCR analysis of the expression levels of differentially functional genes and transcription factors in T1-T4. (a-e) The relative expression levels of five functional genes. (f-l) The relative expression levels of seven transcription factor. Data are presented as mean ±SE, n=3 biologically independent samples; statistical significance was calculated by one-way ANOVA with multiple comparisons between adjacent stages, and was indicated by asterisks, in which n.s. (no significance), $p < 0.05$ (*), $p < 0.01$ (**), $p < 0.001$ (***), and $p < 0.0001$ (****).

Supplementary Tables

Supplementary Table 1. Statistics of the ONT data.

Type	Reads	
	Length (bp)	Number
Max length	192,214	-
N10	49,355	408,698
N20	41,320	937,430
N30	35,900	1,554,656
N40	31,554	2,260,153
N50	27,783	3,061,607
N60	24,305	3,974,310
N70	20,919	5,025,187
N80	17,383	6,265,547
N90	13,061	7,821,155
Total length	237,192,581,205	-
GC rate	10,834,388	-

Supplementary Table 2. Statistics of *K*-mer analysis.

K-mer value	K-mer number	Genome size (bp)	Repeat (%)	Heterozygosity ratio (%)	Used bases (bp)	Sequence depth (X)
17	40,319,141,267	1,141,851,514	73.19	1.88	45,498,857,936	39.85
25	38,136,922,937	1,159,544,672	51.79	1.92	45,447,333,271	39.19
31	36,392,043,675	1,155,976,467	45.89	1.82	45,452,948,981	39.32

Supplementary Table 3. Statistics of the DNB sequencing and Hi-C data.

Title	Clean read	Clean base	Read Length(bp)	Q20	GC_rate
**DNB sequencing**	304,325,050	45,648,757,500	150	95.00%	36.20%
**Hi-C**	224,256,200	33,638,430,000	150	97.67%	36.76%
	804,495,060	120,674,259,000	150	96.36%	36.43%

Supplementary Table 4. Statistics of preliminary assemblies.

Type	Contigs	
	Length (bp)	Number
Max length	14,707,921	-
N10	9,859,925	11
N20	7,617,612	24
N30	6,296,687	40
N40	5,019,884	60
N50	3,964,735	85
N60	3,312,207	116
N70	2,772,300	152
N80	1,917,446	201
N90	1,308,821	271
Total length	1,118,545,505	-
GC rate	0.345	-

Supplementary Table 5. Statistics of final assemblies.

	Contig		Scaffold	
	Length (bp)	Number	Length (bp)	Number
**Longest length**	12,690,304	-	69,209,139	-
**N10**	9,899,931	10	57,888,713	2
**N20**	7,494,773	23	52,145,335	4
**N30**	5,948,291	39	48,312,859	6
**N40**	4,930,051	59	46,838,504	8
**N50**	3,956,172	83	46,330,164	11
**N60**	3,249,569	112	44,138,320	13
**N70**	2,624,073	147	42,598,322	15
**N80**	1,917,446	195	41,625,037	18
**N90**	1,310,726	260	37,379,505	21
**Total length**	1,054,196,928	-	1,054,406,928	-
**Number**	443	-	23	-

Supplementary Table 6. Chromosome length in the final lilac genome assembly.

Pseudochromsomes	Length (bp)
Chr1	69,209,139
Chr2	57,888,713
Chr3	55,872,685
Chr4	52,145,335
Chr5	48,952,543
Chr6	48,312,859
Chr7	47,024,854
Chr8	46,838,504
Chr9	46,449,719
Chr10	46,381,582
Chr11	46,330,164
Chr12	44,258,314
Chr13	44,138,320
Chr14	43,316,361
Chr15	42,598,322
Chr16	42,308,671
Chr17	42,114,989
Chr18	41,625,037
Chr19	41,506,110
Chr20	38,361,950
Chr21	37,379,505
Chr22	37,102,836
Chr23	34,290,416
Total	1,054,406,928

Supplementary Table 7. Summary of BUSCO evaluation results for final assemblies.

Type	Number	Percentage
Complete BUSCOs	1559	96.60%
Complete and single-copy BUSCOs	1360	84.30%
Complete and duplicated BUSCOs	199	12.30%
Fragmented BUSCOs	27	1.70%
Missing BUSCOs	28	1.70%
Total	1614	-

Supplementary Table 8. Summary of the annotated repeat elements in the genome assembly.

Type		Length (bp)	Genome Ratio (%)
**Class I: Retrotransposon**		409,852,575	38.87
	**LTR-Retrotransposon**	398,436,556	37.79
	LTR/Copia	132,260,921	12.54
	LTR/Gypsy	246,386,643	23.37
	LTR-other	19,788,992	1.88
	**Non-LTR Retrotransposon**	11,416,019	1.08
	SINE	85,923	0.01
	LINE	11,330,096	1.07
**Class II: DNA Transposon**		53,103,240	5.04
	EnSpm/CACTA	19,979,025	1.89
	Harbinger	2,923,203	0.28
	Helitron	7,698,862	0.73
	MuDR	9,743,431	0.92
	Tcl/Mariner	1,300,499	0.12
	hAT	10,463,818	0.99
	DNA-other	994,402	0.09
**Low Complexity**		3,628,112	0.34
**Tandem repeat**		39,144,238	3.71
**Unclassified**		66,765,933	6.33
**Total content**		572,494,098	54.30

Note: LTR: Long Terminal Repeat retrotransposons; SINE: Short Interspersed Nuclear Element, a category of non-autonomous and non-coding retroelements (TEs); LINE: Long Interspersed Nuclear Element, a category of non-LTR (long terminal repeat) retroelements.

Supplementary Table 9. Annotation of the predicted lilac genes.

Annotation	Number/Size
Number of predicted genes	35,313
Gene length_average/median (bp)	4,515.34/3,336
Gene length_maximum/minimum (bp)	55,963/303
Number of mRNA	44,281
mRNA length_average/median (bp)	1,650.36/1,409
mRNA length_maximum/minimum (bp)	16,640/300
Number of CDS	44,281
CDS length_average/median (bp)	1,148.13/957
CDS length_maximum/minimum (bp)	16,353/297
Exon length_average/median (bp)	257.32/149
Number of exon_average/median	6.41/5
Intron length_average/median (bp)	628.31/245
Number of intron_average/median	5.41/4
3′-UTR_average/median (bp)	277.49/225
5′-UTR_average/median (bp)	191.86/130
Number of predicted protein-coding genes	33,268 (94.21%)
Annotated to COG	30,454 (86.24%)
Annotated to GO	13,431 (38.03%)
Annotated to KEGG	15,733 (44.55%)
Annotated to NR	33,242 (94.14%)
Annotated to Swiss-Prot	25,882 (73.29%)
Annotated to MSU Rice (*Oryza sativa*)	29,833 (84.48%)
Annotated to Tair (*Arabidopsis thaliana*)	29,765 (84.29%)

Supplementary Table 10. Statistics of the predicted genes integrity.

Type	Number	Percent (%)
Both	32,408	91.77
Only Start	627	1.78
Only End	2,130	6.03
None	148	0.42
Total	35,313	100.00

Supplementary Table 11. Summary of RNAs in the lilac genome annotation.

Type		Number	Average length (bp)	Total length (bp)	Percentage of Genome (%)
miRNA		231	120	27,667	0.0030%
tRNA		724	74	53,509	0.0050%
	rRNA	138	189	26,119	0.0020%
rRNA	18S	19	630	11,970	0.0010%
	28S	5	196	982	0.0000%
	5.8S	8	93	745	0.0000%
	5S	106	117	12,422	0.0010%
	snRNA	3,690	126	131,233	0.0120%
snRNA	CD-box	3,462	105	362,213	0.0340%
	HACA-box	93	129	11,976	0.0010%
	splicing	135	145	19,511	0.0020%

Supplementary Table 12. Genomic data used for comparative genome analysis.

Family	Species	Links
Oleaceae	*Olea europaea*	http://olivegenome.org/downloads/
Oleaceae	*Fraxinus excelsior*	http://ashgenome.org/data
Oleaceae	*Osmanthus fragrans*	http://117.78.20.255/download
Brassicaceae	*Arabidopsis thaliana*	https://phytozome.jgi.doe.gov/pz/portal.html#!info?alias=Org_Athaliana_er
Caricaceae	*Carica papaya*	https://phytozome.jgi.doe.gov/pz/portal.html#!info?alias=Org_Cpapaya
Rosaceae	*Prunus mume*	https://github.com/lileiting/prunusmumegenome
Vitaceae	*Vitis vinifera*	https://phytozome.jgi.doe.gov/pz/portal.html#!info?alias=Org_Vvinifera
Ericaceae	*Rhododendron delavayi*	https://www.researchgate.net/publication/319309848_The_draft_genome_assembly_of_Rhododendron_delavayi_Franch_var_delavayi
Solanaceae	*Solanum lycopersicum*	https://phytozome.jgi.doe.gov/pz/portal.html#!info?alias=Org_Slycopersicum
Solanaceae	*Solanum tuberosum*	ftp://ftp.ncbi.nlm.nih.gov/genomes/Solanum_tuberosum/
Gramineae	*Oryza sativa*	https://genome.jgi.doe.gov/portal/pages/dynamicOrganismDownload.jsf?organism=Osativa

Supplementary Table 13. Summary of gene family analyses for lilac and 11 reference plant species.

Type	*S. oblata*	*A. thaliana*	*C. papaya*	*F. excelsior*	*O. europaea*	*O. sativa*	*O. fragrans*	*P. mume*	*R. delavayi*	*S. lycopersicum*	*S. tuberosum*	*V. vinifera*
Number of genes	35,313	27,654	27,775	38,949	50,684	39,049	45,490	31,390	32,938	35,768	35,119	31,845
Number of genes in orthogroups	33,058	25,183	23,150	36,436	46,190	30,175	42,958	28,800	29,668	31,175	32,718	27,409
Number of unassigned genes	2,255	2,471	4,625	2,513	4,494	8,874	2,532	2,590	3,270	4,593	2,401	4,436
Percentage of genes in orthogroups (%)	93.6 (33,058/35,313)	91.1	83.3	93.5	91.1	77.3	94.4	91.7	90.1	87.2	93.2	86.1
Percentage of unassigned genes (%)	6.4 (2,255/35,313)	8.9	16.7	6.5	8.9	22.7	5.6	8.3	9.9	12.8	6.8	13.9
Number of orthogroups containing species	14,829	13,154	13,804	15,520	15,651	13,726	16,139	14,362	14,390	15,745	14,497	14,852
Percentage of orthogroups containing species (%)	49.7 (14,829/29,856)	44.1	46.2	52	52.4	46	54.1	48.1	48.2	52.7	48.6	49.7
Number of species-specific orthogroups	400	756	531	242	662	2,225	764	867	989	533	481	727

Supplementary Table 14. Summary of intra- and inter- genomic collinearity.

Species	Intra-genome/Inter-genome	
	Blocks Number	Gene Pairs Number
S. oblata	855	15,786
Osmanthus fragrans	1,403	28,652
S. oblata - O. fragrans	532	34,576
S. oblata - V. vinifera	1,562	15,803

Supplementary Table 15. Summary of significantly differential metabolites.

Group name	All sig diff	Down regulated	Up regulated
T1vsT2	39	20	19
T1vsT3	43	29	14
T1vsT4	46	31	15
T2vsT3	30	18	12
T2vsT4	38	20	18
T3vsT4	37	17	20

Supplementary Table 16. Summary of differentially expressed genes (DEGs).

Group name	All DEGs	Down regulated	Up regulated
T1vsT2	23,568	11,893	11,675
T1vsT3	20,011	10,405	9,606
T1vsT4	19,158	9,435	9,723
T2vsT3	17,789	9,042	8,747
T2vsT4	22,910	11,294	11,616
T3vsT4	15,133	7,059	8,074

Supplementary Table 17. Primers used in qRT-PCR.

Gene	Forward (5' to 3')	Reverse (3' to 5')
F3'H_So01G039330	AGCACAAGATCAACGGCTCT	TGCGCAACATCTTTCAACTC
F3'H_So05G012500	TTTTGCAGCGTTTTGATCTG	CCTTGTTCTCTGCCCCATTA
F3'H_So07G050530	GGACTTGTTGAGTGCGTTGA	TCTCTGGGTGTCGAAGGAGT
CHS_So19G016880	CAAAATCACCCACCTCGTCT	AAGCAGCCCTGCTGATACAT
F3H_So17G006510	AGCTCCGATTCAACCATCAC	GGGGATCGTCGTAATAAGCA
ERF_So11G003650	GAATGCCAAAACGAACTTCC	TTTCGCAACTTTGCACTGAG
ERF_So15G039730	AGGATCTGCAAACCGTCAAC	TTGGCCTTGGAATGTTCTTC
GATA_So01G024070	TCCAATGGTTTTTCCGAGAC	GGAAGAGACCCGAAATCCTC
WD40_So01G077230	CAAGAAATTAGCGCACACGA	CCCCATCTTCACCTCCACTA
bHLH_So17G021180	TGAAGGGAAATGGGATTTTG	ACCGTGTTGGCAAATTCTTC
bHLH_So09G026360	CGCCTTTATCAGCAACAACA	TGGCAAATTGATTTCATCCA
bHLH_So03G051480	TGCAGTACCTCCAAGGGAAG	AGGATTTCTGGATCGACACG
Soactin	TGTGCTTTCCCTCTACGCCAGT	TCCCTCACGATTTCCCGCTCT
