# Supplementary material for: Lilac (Syringa oblata) genome provides insights into its evolution and molecular mechanism of petal color change
Source: Commun Biol. 2022 Jul 9;5:686. doi: 10.1038/s42003-022-03646-9 (PMC9271065; doi:10.1038/s42003-022-03646-9)
Supplement: Supplementary file 3 — Description of Additional Supplementary Files [file 42003_2022_3646_MOESM3_ESM.pdf]

Description of Additional Supplementary Files

File name: Supplementary Data 1
Description: Summary of 118 flavonoid metabolites detected based on UPLC-MS/MS.

File name: Supplementary Data 2
Description: The source data behind the Figure 4b in the paper.
